# Role of RNA Binding Proteins with prion-like domains in muscle and neuromuscular diseases

**DOI:** 10.15698/cst2020.04.217

**Published:** 2020-03-10

**Authors:** Gina Picchiarelli, Luc Dupuis

**Affiliations:** 1 Université de Strasbourg, INSERM, Mécanismes Centraux et Périphériques de la Neurodégénérescence, UMR_S 1118, Strasbourg, France.

**Keywords:** RNA-Binding Protein (RBP), Muscle, Dystrophy, Amyotrophic lateral sclerosis (ALS), Spinal muscular atrophy (SMA), Inclusion body myopathy (IBM), Fragile X-associated tremor / ataxia syndrome (FXTAS), Multisystem proteinopathy (MSP), Huntington's disease

## Abstract

A number of neuromuscular and muscular diseases, including amyotrophic lateral sclerosis (ALS), spinal muscular atrophy (SMA) and several myopathies, are associated to mutations in related RNA-binding proteins (RBPs), including TDP-43, FUS, MATR3 or hnRNPA1/B2. These proteins harbor similar modular primary sequence with RNA binding motifs and low complexity domains, that enables them to phase separate and create liquid microdomains. These RBPs have been shown to critically regulate multiple events of RNA lifecycle, including transcriptional events, splicing and RNA trafficking and sequestration. Here, we review the roles of these disease-related RBPs in muscle and motor neurons, and how their dysfunction in these cell types might contribute to disease.

## INTRODUCTION

**Key Concepts:**
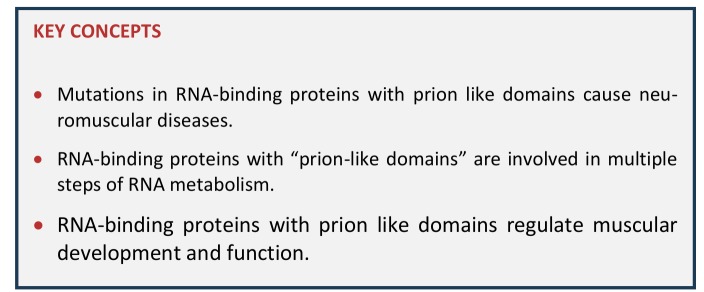
KEY CONCEPTS

Neuromuscular diseases collectively affect muscle function, either by directly impairing muscle structure or function, or by affecting muscle control by motor neurons. As a consequence of impaired muscle function, patients develop weakness that can be progressive and lead to paralysis and early death. Amyotrophic lateral sclerosis (ALS) and spinal muscular atrophy (SMA) are two typical diseases of the motor neurons, in which muscle weakness is primarily caused by the degeneration of motor neurons [1]. In contrast, myopathies primarily affect muscle structure and/or function with clinically affected muscles either proximal, such as in limb girdle muscle dystrophy, and/or distal in distal myopathies. Although the distinction between primary muscle and primary neuronal neuromuscular diseases might a priori seem obvious, there are significant clinical and genetic overlaps between these diseases [2–4]. In this review, we describe how mutations in functionally related RNA-binding proteins (RBPs) are associated with both muscle and motor neuron diseases, and how these mutations participate in compromising the neuromuscular system. The most important neuromuscular diseases considered are presented in **BOX 1**.

**Box 1:**
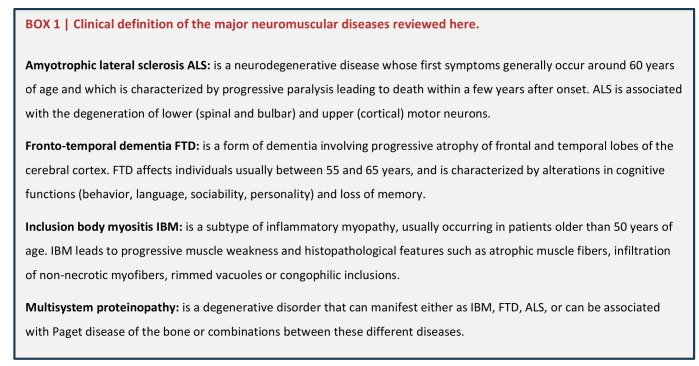
BOX 1: Clinical definition of the major neuromuscular diseases reviewed here.

In recent years, genetics uncovered a large number of causes of neuromuscular diseases. Interestingly, a subset of genes causing either motor neuron diseases or myopathies encode proteins that bind RNA (hence RNA-binding proteins, RBPs) and share a number of biochemical and functional properties. RBPs associated to neuromuscular diseases are part of a large group of proteins involved in mRNAs lifecycle, that are collectively termed heterogeneous nuclear ribonucleoproteins (hnRNPs). Many of these hnRNPs also display a low complexity domain that resembles yeast prions and is called “prion-like domain” (PrLD). Most of these PrLD containing RBPs are associated with human diseases [5–9], in particular neuromuscular diseases.

In this review, we describe the general properties of disease associated RBPs. We then provide specific examples for the involvement of RBPs in neuromuscular diseases.

## MODULAR STRUCTURE AND GENERAL CELLULAR FUNCTIONS OF RBPs

**Figure 1:**
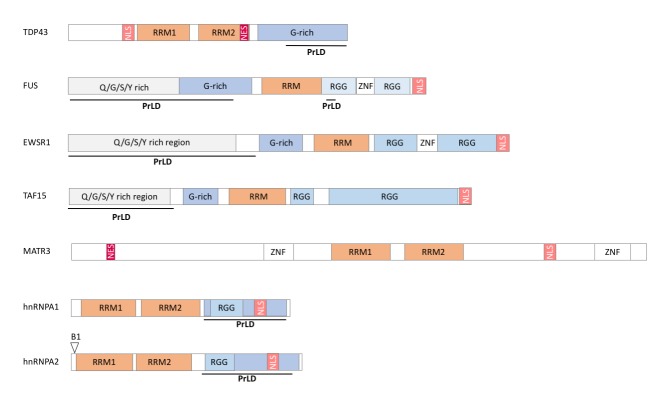
FIGURE 1: Domain organization of RBPs with prion like domains. NLS: nuclear localization signal, NES: nuclear export signal, RRM: RNA recognition motif, RGG:arginine/glycine-rich region, G-rich: glycine-rich region, ZNF: Zinc finger motif, Q/G/S/Y rich region: glutamine, glycine, serine and tyrosine-rich region, B1: B1 isoform of hnRNPA2, PrLD : prion like domain.

RBPs associated to neuromuscular diseases display a modular structure with well identified subdomains. First, interaction of RBPs with RNA occurs through relatively limited sets of protein modules [10] in particular RNA recognition domains (RRM) and/or hnRNPK homology domain (KH). Other protein domains are variably present in RBPs and include Arginine-Glycine-Glycine rich domain (RGG), double-stranded RNA binding motifs (dsRBM), DEAD box, A2 recognition element (A2RE), AU rich element (ARE), Zinc fingers domain (Zn), Zn-knuckle motifs, S1 domain, PAZ and PIWI domains [10, 11]. **(Figure 1)**.

This interaction with various RNA species, as well as their capacity to shuttle between nucleus and cytoplasm allow RBPs to participate in all steps of the mRNA cycle, from transcription, maturation, transport, translation, stability to degradation [11–14]. RBPs also contribute to translational and post translational regulation through binding to 3' untranslated regions (UTR) of mRNAs [15]. Besides mRNAs, a number of these RBPs are also critical in the life cycle of small RNA species, in particular microRNA biogenesis [16–18].

The so-called PrLD is typically found in most disease associated RBPs. It consists of a domain of low primary sequence complexity, rich in uncharged polar amino acids (asparagine, glutamine, and tyrosine) and in glycine [19, 20] and displays high similarity to yeast proteins with prion properties [21]. In the human genome, more than 200 encoded proteins display a PrLD, and a large proportion of these also include RNA binding motifs [14, 19, 20]. The combination of RNA binding properties with PrLDs allows RBPs to phase separate in liquid compartments. Liquid Liquid Phase Separation (LLPS) is a disassembly mechanism of two liquids resulting in the appearance of two phases [22]. This leads to the rapid and reversible creation of liquid microdomains (so called membrane-less organelles), physically separated from the rest of the cell, and allowing specialized functions. [14]. In this respect, RBPs are required for the generation and maintenance of key nuclear subdomains such as nucleoli, paraspeckles, gems, Cajal bodies, P-bodies or cytoplasmic stress granules through LLPS. Disease associated mutations in RBPs compromise LLPS, leading to the appearance of solids aggregates [23–26].

In the next sections, major RBPs are reviewed for their involvement in neuromuscular diseases **(Table 1)**.

**TABLE 1.** Summary of selected RBP with prion like domain in neuromuscular disease.

RBP	Reported RNA motifs	Functions in muscle	Pathological alterations	RBP-associated muscular disease
MATR3	UC-rich motif [136, 176]	Proliferation	Mutations	VCPDM
		Differentiation	Aggregates	ALS
hnRNP	UAGG motifs [177]	Muscle development	Mutations	FXTAS
		Contraction	Aggregates	ALS
				FTD
				LGMD1
				OPMD
				MP
				SMA
TDP43	(GU)_n_ repeat	Muscle development	Mutations	ALS
	UG motifs [178, 179]	NMJ formation	Aggregates	FTD
		Mitochondrial functions		MD
				IBM
				SMA
FUS	Several motifs reported, including GGUG, GU-rich and CU rich hexamers [170, 180–184]	Muscle development	Mutations	SMA
		Differentiation	Aggregates	ALS
		NMJ formation		FTD
		Mitochondrial functions		MG
				HD
EWSR1	G-rich motif [181]	Muscle development	Mutations	ALS
		Differentiation	Aggregates	FTD
		Proliferation		SMA
		Mitochondrial functions		
TAF15	GGUAAGU [181, 185]	Mitochondrial fusion	Mutations	ALS
			Aggregates	FTD

ALS: Amyotrophic lateral sclerosis, DM: Distal myopathy, FXTAS: Fragile X-associated tremor/ataxia syndrome, HD: Huntington disease, IBM: Inclusion body myopathy, LGMD1: limb-girdle muscular dystrophy 1D, MD: Muscular dystrophy, MG: Myasthenia gravis, MP: Multisystem proteinopathy, OPMD: Oculopharyngeal muscular dystrophy, SMA: Spinal muscular atrophy, VCPDM: Vocal cord and pharyngeal weakness with distal myopathy.

## TDP43

TAR DNA-binding protein of 43 kDa (TDP43) is an RBP able to bind to single stranded DNA and RNA in order to modulate splicing, RNA stability and biogenesis [27–29]. TDP43 was initially characterized as a protein binding to the retroviral protein Tar [30] and later shown to modulate the splicing of key exon 9 in the *CFTR* gene associated with cystic fibrosis [28]. In 2006, a landmark study identified TDP43 protein as the major ubiquinated protein in aggregates present in patients with ALS and fronto-temporal dementia (FTD), two major neurodegenerative diseases **(BOX 1)** [31]. Indeed, TDP43 inclusions have been found in approximatively 95% of all ALS cases (sporadic and familial) and half of the FTD cases [31]. Subsequently, mutations in the *TARDBP* gene, encoding TDP43, were found to account for 3% of familial cases and 1.5% of sporadic cases of ALS [32–34].

How TDP43 aggregates are linked to neurodegeneration in ALS and FTD is complex and still not completely understood. First, TDP43 aggregates are cytoplasmic and associated with complete nuclear clearance of TDP43 [35], and cells with TDP43 aggregates thus display loss of TDP43 nuclear function. Indeed, loss of function of TDP43 in motor neurons is sufficient to trigger motor neuron degeneration [36–38], that is likely due to defective repression of splicing of cryptic exons [39, 40] and defective autophagy [38]. Gain of function mechanisms are also likely to participate as expression at physiological levels of mutant TDP43 is able to drive neurodegeneration [41–45]. It is likely that the function of TDP43 in splicing in motor neurons is critical in this mutant gain of function [43, 45, 46]. A potential critical target is the *Tardbp* mRNA (encoding TDP43) itself whose autoregulation is disrupted upon the expression of a mutant TDP43 [43, 45]. Altered TDP43 function might also be involved in other motor neuron diseases, such as SMA. In this disease, caused by loss of the survival of motor neurons (SMN) protein, TDP43 might contribute to the splicing dysfunction caused by loss of SMN. Indeed, TDP43 promotes the inclusion of exon 7 of the SMN2 pre-mRNA *in vitro* [47] and depletion of TDP43 leads to reduction and loss of gems, thereby strengthening the role of TDP43 in SMA [48–50]. Thus, TDP43 might participate directly or indirectly in the pathophysiology of a number of neurodegenerative disorders.

**Figure 2:**
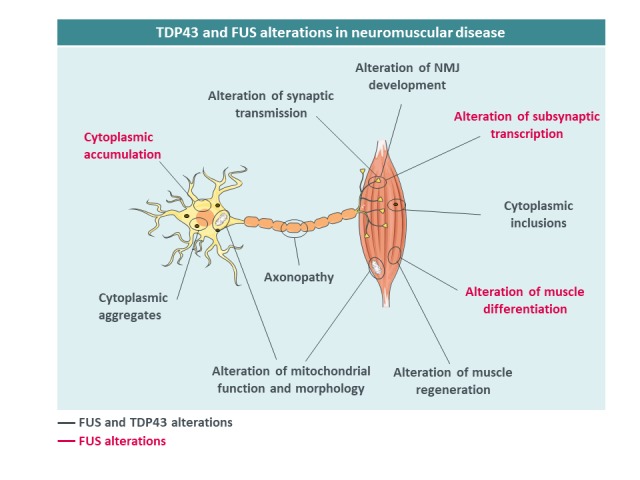
FIGURE 2: TDP43 and FUS alterations in neuromuscular diseases. Mutant TDP43 aggregates are found in motor neurons and muscles of patients. TDP-43 mislocalization causes axonopathy and mitochondrial alterations, alters synaptic transmission, NMJ development and muscle regeneration. Similar observations are made for FUS, which is also involved in subsynaptic transcription.

Beyond neurons, TDP43 has been shown to be critical for skeletal muscle function, pointing towards a potential involvement of TDP43 in muscle diseases. TDP43 is required for muscle regeneration [51] and forms cytoplasmic granules sequestering sarcomeric RNAs to facilitate regeneration. Furthermore, TDP43 is required for expression of critical regulators of myogenesis such as MYOD or MYOG [52] and key myogenic microRNAs such as miR-1 and 206 [53]. Consistently, TDP43 loss of function [54, 55] or muscle overexpression of TDP43 is highly detrimental for muscle structure and function [56, 57]. TDP43 also participates in neuromuscular junction (NMJ) formation at least in Drosophila [58, 59]. This importance of TDP43 in muscle function indirectly suggests that this protein could be involved in muscle dysfunction in human diseases. Indeed, muscle cytoplasmic aggregates of TDP43 were observed in patients with ALS, muscle dystrophy and inclusion body myositis (IBM) [60–68]. TDP43 might also indirectly participate in muscle pathology developed during inherited peripheral neuropathies of myofibrillar myopathies [69] **(Figure 2)**.

## FUS

FUS is an RBP belonging to the FET family, that also includes EWS and TAF15. The FET proteins are predominantly localized in the nucleus where they control DNA/RNA metabolism [70, 71]. Multiple results demonstrate a pleiotropic function of FUS in regulating mRNA expression, stability, maturation in multiples cells including muscle cells.

Mutations in the *FUS* gene have been identified in patients with ALS in 2009 [72, 73] and currently more than 50 mutations in this gene have been described. ALS patients with *FUS* mutations show generally an earlier age at onset, sometimes in their 20's, and aggressive progression [74]. Most of these mutations are in or around the C-terminal nuclear localization signal (NLS) [73, 75–78], and severity is correlated with the degree of impairment of FUS nuclear import [76]. FUS aggregates are also found in a subset of FTD patients, yet in the absence of germline mutations, with different post-translational modifications [79, 80] and with co-deposition of other proteins including TAF15, EWS and TNPO1 [81–83]. In a manner similar to TDP43, both gain and loss of FUS function have been postulated to participate in FUS-related neurodegeneration. First, FUS cytoplasmic accumulation, due to loss of nuclear import, might lead to neuronal death through a so-called cytoplasmic gain of function. In particular, cytoplasmic FUS might sequester proteins of importance, such as SMN [49, 84–87] or PRMT1 [88, 89] and lead to the accumulation of toxic stress granules and cytoplasmic aggregates [79, 88, 90]. Second, clearance of FUS from the nucleus might lead to alterations in the many nuclear FUS functions, including transcription, splicing or DNA damage repair [91, 92]. Importantly, accumulation of cytoplasmic FUS is necessary to lead to motor neuron degeneration in mice [93–96]. For instance, we and others have shown that heterozygous *Fus* knock-in mouse models with truncated mutations develop mild, late onset muscle weakness and motor neuron degeneration, but not haploinsufficient *Fus* knock-out mice, demonstrating that the presence of the protein in the cytoplasm is necessary to trigger motor neuron toxicity [93, 94, 97]. Loss of FUS function might contribute to FTD symptoms, through alterations of splicing of key neuronal mRNAs such as *MAPT,* encoding the TAU protein, or of stability of mRNAs encoding synaptic proteins such as GluA1 and SynGAP1 [98–102]. Although less studied than its function in neurons, FUS plays important roles in the muscle. Indeed, muscles of sporadic IBM [64, 65] can display FUS aggregates, while mutations in *FUS* were found in one patient with myositis [103]. In the muscle, FUS regulates alternative splicing and differentiation through its action on DUX4 and PTBP1 [104, 105] but also hnRNPA1 and MATR3, two proteins involved in muscle development [106] and ALS [20, 107, 108]. Furthermore, FUS has been shown to be important for the function of PGC1α, a key regulator of muscle mitochondrial function [109]. FUS also exerts critical roles in neuromuscular junction development. Animal models of FUS-ALS show alteration of synaptic transmission and modification of NMJ numbers and size [110–114], and we recently demonstrated that FUS is required for the post-synaptic development of the NMJ [115]. Indeed, both knock-in and knock-out mice for *Fus* developed NMJ morphology defects. Newborn homozygous *Fus* mutant mice displayed predominantly postsynaptic NMJ defects whereas adult heterozygous Fus mutant mice displayed constitutively smaller neuromuscular endplates that denervate. Importantly, FUS was enriched in muscular subsynaptic nuclei and this enrichment depended on innervation and was perturbed in heterozygous *Fus* mutant mice. Mechanistically, FUS binds to the promoter region and stimulates transcription of acetylcholine receptor (AchR) subunit genes involved in NMJ formation through the transcription factor ERM. In induced pluripotent stem cell (iPSC)-derived myotube cultures and motor neuron/myotube co-cultures from FUS-ALS patients, endplate maturation was impaired and AChR expression reduced. Finally, in motor neuron/myotube co-cultures, ALS-mutant FUS was intrinsically toxic to both motor neurons and myotubes. Altogether, these data show that FUS plays a key role in regulating selective expression of AChR genes in subsynaptic nuclei and indicate that intrinsic toxicity of ALS-mutant FUS in the muscle may be critical for ALS [115].

FUS is also involved in SMA, through a direct interaction between FUS and SMN through the U1-snRNP. Similar as TDP43, FUS is associated with gems, that are affected by ALS causing mutations [49, 113]. Furthermore, snRNAs seem to be trapped by cytoplasmic FUS [116, 117].

Besides motor neuron diseases, FUS is also associated with other neuromuscular diseases such as myasthenia gravis and Huntington's disease. Myasthenia gravis is an autoimmune disorder of the NMJ inducing skeletal muscle weakness. In this disease, an increase of *FUS* transcript is observed in the blood of myasthenic patients [118]. Its significance remains unknown. In Huntington's disease, mutant huntingtin (HTT) protein sequesters FUS in neuronal inclusions [99, 119, 120].

## EWS

EWS is the second member of the FET family, encoded by the *EWSR1* gene. This oncogenic protein is involved in proliferation and cell differentiation [121–123]. In analogy with *FUS* mutations, Couthouis and collaborators identified three *EWSR1* missense mutations in ALS patients able to lead to EWS mislocalization in the cytoplasm of motor neurons. Indeed, EWS appears to be mislocalized in the cytoplasm of motor neurons in sporadic ALS in the absence of EWS mutations [19, 124] and FUS-FTD [81]. Like for FUS and TDP43, EWS interacts with SMN and is required for its function in splicing, suggesting a role of EWS in SMA [125, 126].

In muscle, EWS may participate to myogenesis through its regulation of the transcriptional co-activator PGC1α. Indeed, EWS loss leads to PGC1α degradation due to impaired stability [127]. Consistently, the loss of *EWSR1* causes abnormalities in mitochondrial structure and a decrease in DNA and mitochondrial density.

## TAF15

TAF15, the last member of FET family, shares similar structure and functions as FUS and EWS and appears associated with ALS.

In 2011, Couthouis *et al.* identified three missenses mutations in TAF15, whereas Ticozzi *et al.* discovered four other mutations in ALS patients [19, 128]. These mutations affect mainly the RGG domain [128] and promote cytoplasmic foci in primary rat embryonic neuron cultures [19]. In human post-mortem spinal cord tissue of control patients TAF15 is nuclear while TAF15 in ALS patients is nuclear and forms cytoplasmic aggregates. Furthermore, neurodegeneration and abnormal mitochondrial fragmentation in muscle and motor neurons were observed in TAF15 ALS fly models [19, 129]. These mitochondrial abnormalities are mediated by mitofusins as mutant TAF15 decreases mitofusin protein expression and mitochondrial defects can be rescued upon rescue of mitofusin in *Taf15* mutant flies [129].

## MATR3

Matrin 3 (MATR3) is a 125 kDA nuclear matrix protein [130] of 845 amino acids [131]. MATR3 binds and stabilizes RNA [132] in multiple tissues especially skeletal muscle. Contrary to previous examples, MATR3 has no prion-like domain per se, but several intrinsically disordered regions.

In myotubes, MATR3 is present in the nuclear matrix and nuclear membrane [133] and its localization is dependent upon the expression of the muscle specific transcription factor Myogenin [133].

MATR3 has been found to be critical in multiple gene expression events related to muscle function and differentiation. First, MATR3 is required for normal myoblast proliferation and differentiation since its overexpression increases the expression of myogenic related genes [134]. Conversely, MATR3 depletion decreases protein levels of myogenin and decreases the differentiation status. MATR3 regulates alternative splicing through its interaction with the Polypyrimidine Tract Binding Protein (PTBP) that is critical in muscle differentiation [135, 136]. Furthermore, MATR3 binds to and regulates long non-coding RNA in muscles [134]. Last, MATR3 binds directly to Lamin A, a protein required for muscle differentiation [133, 137, 138]. Interestingly, mutations in LMNA gene encoding lamin A/C lead to skeletal and cardiac myopathy [139] and disrupt lamin A/MATR3 interaction [133].

Mutations in *MATR3* have been first associated with muscular diseases. First, a *MATR3* missense mutation p.Ser85Cys (chr5:138643358, C>G) was associated with vocal cord and pharyngeal weakness with distal myopathy (weakness and atrophy of the hands and feet) [140, 141]. The distal myopathy associated with *MATR3* mutation usually begins within the fourth decade, and is characterized by heterogeneous involvement of distal limb muscles, pharyngeal and respiratory muscles, leading to proximal and axial weakness, vocal cord dysfunction with mild voice abnormalities, dysphagia and decreased respiratory function [141–144].

More recently, mutations in *MATR3* have been associated with ALS. Johnson *et al.* performed exome sequencing and identified novel missense mutations associated with ALS in *MATR3*: p.Phe115Cys (chr5:138643448, T>G) and p.Thr622Ala (chr5:138658372, A>G) [107]. Interestingly, the p.Phe115Cys mutation caused a respiratory form of ALS leading to death within five years of symptom onset whereas the p.Ser85Cys mutation (identified in distal myopathy) induced a slowly progressive form of ALS. MATR3 immunostaining showed a partial mislocalization in the cytoplasm of motor neurons and surrounding glial cells in ALS patients but no cytoplasmic inclusions were observed. MATR3 and TDP43 co-aggregated in skeletal muscles of patients and a direct interaction was observed between MATR3 and TDP43, another RBP linked to ALS. Recently a novel missense mutation p.Ser610Phe was discovered in one patient and three missense variants p.Ala313Gly, p.Arg147Lys, and p.Gln347Lys were observed in three healthy subjects [145]. Thus, similar to several other RBPs, mutations in *MATR3*, can lead to a broad spectrum of neuromuscular diseases, from pure muscle involvement to severe motor neuron disease.

## OTHER hnRNPs

A number of other hnRNPs has been associated with various neuromuscular diseases.

First, hnRNPA3 was found to bind to mutant *C9ORF72* RNA in ALS and could mediate some of its toxic effects [146, 147]. Furthermore, hnRNPA3 was also reported to be present in TDP43, p62 immunoreactive dipeptide repeat (DPR) inclusions in C9orf72 cases [148, 149] further linking hnRNPA3 to C9orf72 ALS/FTD. Second, mutations in hnRNPA1 and hnRNPA2B1 have been identified in multisystem proteinopathy, a disorder combining IBM, FTD, ALS or Paget's disease of the bone (PDB) [20]. Disease mutations impact C-terminal regions of hnRNPA2 (residues 185–341) and hnRNPA1 (residues 186–320) which are located in the PrLD, essential for RNA granule formation. Indeed, disease associated mutations of hnRNPA2B1 and hnRNPA1 alter stress granule formation through cytoplamic mislocalization and accelerated fibrillization of the mutant protein. Interestingly hnRNPA1 and hnRNPA2B1 co-localize in stress granule with TDP43 and VCP, two proteins involved in ALS. hnRNAPA1 could be involved in ALS and was shown to be mislocalized in post-mortem samples of ALS patients [150]. Moreover, hnRNPA1 interacts and co-localizes with wild type but not mutant FUS.

hnRNPA2B1 could be involved in Fragile X-associated tremor/ataxia syndrome (FXTAS), a late onset disorder inducing a form of mental retardation. This disease is caused by expansion of more than 200 CGG in the *FMR1* gene and provokes tremor, ataxia and cognitive defects [151]. In 2007, Sofola and collaborators identified an interaction between hnRNPA2/B1 and the mutant RNA carrying CGG repeats in mouse cerebellar lysates [152]. Consistent with RNA toxicity, overexpression of hnRNPA2/B1 prevents the neurodegenerative eye phenotype induced in CGG transgenic flies. In muscles, Liu and collaborators showed that hnRNPA1 depletion causes muscle developmental defects associated with an increase of myofibers in the heart, a decrease in diaphragm and tongue [106] and dysregulated expression of the genes involved in the development and muscular contraction. Indeed, hnRNPA1 and hnRNPA2/B1 are also involved in limb-girdle muscular dystrophy 1D (LGMD1D). This skeletal and cardiac myopathy, can be caused by missense mutations in DNAJB6, induces ambulation problems and is characterized by myofibrillar protein aggregation and autophagic rimmed vacuoles. Recently, Bengoechea and collaborators reported an accumulation and co-localization of hnRNPA1 and hnRNPA2/B1 with DNAJB6 in sarcoplasmic stress granules [67]. Further strengthening the link between hnRNPs and LGMD, hnRNPDL mutations were observed in LGMD1G [153], and are thought to cause disease through aggregation in muscle and loss of function [154].

hnRNPs are also associated with oculopharyngeal muscular dystrophy (OPMD) an adult-onset disease characterized by droopy eyelids, external ophthalmoplegia, dysphagia and proximal limb weakness. OPMD is caused by a GCG repeat expansion in PABPN1 (poly(A)-binding protein N1) and induces inclusions. These contain insoluble intranuclear aggregates of PABPN1 but also hnRNPA1 and A/B [155]. Finally, hnRNPs are also involved SMA, a juvenile neuromuscular disorder characterized by a loss of motor neurons, muscular weakness and wasting. The disease is caused by a mutation in the *SMN1* gene and several studies revealed an interaction between SMN and hnRNPA1, HnRNPC1/C2, hnRNPG, hnRNPM, hnRNPQ, hnRNPR [156–163].

## A NETWORK OF RBPs TO FINE TUNE NEUROMUSCULAR HEALTH

**Figure 3:**
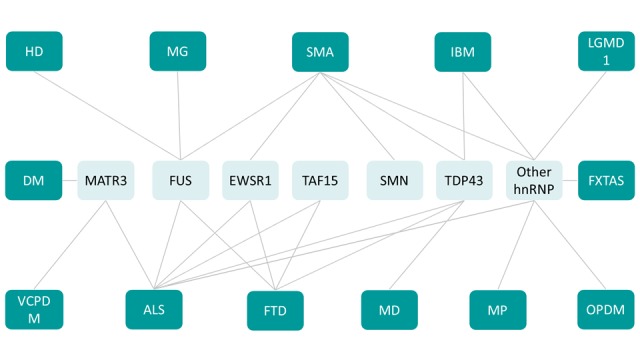
FIGURE 3: RBP with prion like domain network in neuromuscular disease. ALS: Amyotrophic lateral sclerosis, DM: Distal myopathy, FXTAS: Fragile X-associated tremor/ataxia syndrome, HD: Huntington disease, IBM: Inclusion body myopathy, LGMD1: limb-girdle muscular dystrophy 1D, MD: Muscular dystrophy, MG: Myasthenia gravis, MP: Multisystem proteinopathy, OPMD: Oculopharyngeal muscular dystrophy, SMA: Spinal muscular atrophy, VCPDM: Vocal cord and pharyngeal weakness with distal myopathy.

The occurrences of mutations in multiple functionally related RBPs leading to a vast array of neuromuscular diseases suggest that RBPs are involved in a tight network to regulate neuromuscular health **(Figure 3)**. This RBP network is illustrated by the existence of multiple binary protein-protein interactions between RBPs. For instance, MATR3 interacts with TDP43 [132, 164] as well as with a number of splicing regulators including hnRNPK [132] and hnRNPL [165]. MATR3 and FUS interaction is known to regulate splicing and transcription *in vitro* [166], while FUS and TDP43 interaction is modulated by disease associated mutations [164]. In addition, RBPs appear to regulate levels of other RBPs through splicing. A clear example is provided by TDP43-mediated regulation of HNRNPA1 splicing, leading to altered hnRNPA1 content, and subsequent protein aggregation and cellular toxicity [167].

The functions of RBPs are partially overlapping, as exemplified for instance by the common regulation of *MAPT* splicing by FUS and TDP43 [29, 98, 168] or of HDAC6 mRNA [169]. However, this overlap is only partial, and TDP43 and FUS share only a subset of their mRNA targets [170].

Similarly, while FUS, TAF15, EWS and MATR3 are all required for the function of the U1 snRNP/RNA polymerase II complex, they appear to exert distinct, non-overlapping molecular functions in this complex [171].

Thus, mutations or loss of function of one RBP might alter the whole network, and lead to disease. Consistently, a recent study showed that mutation in FUS has an impact on the homeostasis of a number of RBPs, and that the toxicity of FUS mutations could be mitigated by other RBPs [172]. Similar evidence has been published in zebrafish, with epistatic interactions between FUS and TDP43 [173]. In all, RBP homeostasis should be considered globally and a number of secondary consequences on multiple RBPs could be expected from a mutation in one single member.

## CONCLUSIONS

The different examples reviewed here convincingly demonstrate a strong involvement of RBPs in neuromuscular diseases. Importantly, the pathogenic roles of these proteins go far beyond the rare cases associated with germline mutations, as shown by the widespread aggregation of TDP43 or FUS in ALS and FTD.

However, many questions remain open. First, the relative role of loss of nuclear function versus gain of cytoplasmic function remains an open question. Indeed, while it is clear that the cytoplasmic accumulation is necessary for toxicity, it cannot be excluded that associated loss of nuclear function contributes to the toxicity. Furthermore, if cytoplasmic toxicity appears critical, it is unclear whether toxicity of the mutant proteins occur through aggregation or their soluble forms. Indeed, aggregation of these proteins is generally not observed in knock-in animal models, which correlates with a mild phenotype. In general, biophysical properties of these proteins in the cytoplasm remains to be studied.

Most importantly, the identification of critical pathogenic events downstream of RBP mutation or aggregation remains to be done. In this respect, recent studies demonstrated that loss of nuclear TDP43 in motor neurons triggers loss of stathmin 2 in turn possibly responsible of axonal degeneration [174, 175]. The identification of a limited number of critical events downstream RBPs dysfunction could help to identify relevant targets. Importantly, as the toxicity of mutant RBPs extends beyond motor neurons, including muscles or other cell types, it will be necessary to study such critical events in different cell types to better define possible targets either common to several cell types or cell specific. We would like to specifically stress that the mechanisms underlying toxicity in skeletal muscles should be further investigated, especially given the large body of literature reviewed here showing a critical role of RBPs in muscle development, function and pathologies. It is very likely that the extent of RBP involvement in neuromuscular diseases will grow in the next years.

## Abbreviatons:

AchR: – acetylcholine receptor;
ALS: – amyotrophic lateral sclerosis;
FTD: – fronto-temporal dementia;
hnRNP: – heterogenous nuclear ribo-nucleoprotein;
IBM: – Inclusion body myositis;
NMJ: – neuromuscular junction;
OPMD: – oculopharyngeal muscular dystrophy;
PrLD: – prion-like domain;
RBP: – RNA binding protein;
RGG: – Arginine-Glycine-Glycine rich domain;
SMA: – spinal muscular atrophy;
SMN: – survival of motor neurons.
